# The effect of a mass distribution of insecticide-treated nets on insecticide resistance and entomological inoculation rates of *Anopheles gambiae s.l*. in Bandundu City, Democratic Repub`lic of Congo

**DOI:** 10.11604/pamj.2021.40.118.27365

**Published:** 2021-10-25

**Authors:** Emery Metelo-Matubi, Josué Zanga, Guillaume Binene, Nono Mvuama, Solange Ngamukie, Jadis Nkey, Pauline Schopp, Maxwell Bamba, Seth Irish, Jean Nguya-Kalemba-Maniania, Sylvie Fasine, Jonas Nagahuedi, Jean-Jacques Muyembe, Paul Mansiangi

**Affiliations:** 1 Faculté de Médecine, Université de Bandundu, B.P 548 Bandundu-ville, Bandundu, République Démocratique de Congo,; 2 Institut National de Recherche Biomédicale, B.P 1197 KIN 1, Kinshasa, République Démocratique de Congo,; 3 Faculté des Sciences, Département de Biologie, Unité de Recherche Entomologique, B.P 190 KIN XI, Université de Kinshasa, Kinshasa, République Démocratique de Congo,; 4 Faculté de Médecine, Ecole de Santé Publique, Département de Santé Environnementale, B.P 834 KIN XI, Université de Kinshasa, Kinshasa, République Démocratique de Congo,; 5 United States President´s Malaria Initiative and Entomology Branch, Division of Parasitic Diseases and Malaria, Center for Global Health, Centers for Disease Control and Prevention, 1600 Clifton Road NE, Atlanta, GA 30329, USA,; 6 International Centre of Insect Physiology and Ecology, P.O. Box 30772 - 00100 GPO, Nairobi, Kenya

**Keywords:** *Anopheles gambiae*, resistance, insecticide-treated nets, sporozoite rate

## Abstract

**Introduction:**

insecticide-treated nets (ITNs) remain the mainstay of malaria vector control in the Democratic Republic of Congo. However, insecticide resistance of malaria vectors threatens their effectiveness. Entomological inoculation rates and insecticide susceptibility in Anopheles gambiae s.l. were evaluated before and after mass distribution of ITNs in Bandundu City for possible occurrence of resistance.

**Methods:**

a cross-sectional study was conducted from 15^th^ July 2015 to 15^th^ June 2016. Adult mosquitoes were collected using pyrethrum spray catches and human landing catches and identified to species level and tested for the presence of sporozoites. Bioassays were carried out before and after distribution of ITNs to assess the susceptibility of adult mosquitoes to insecticides. Synergist bioassays were also conducted and target site mutations assessed using Polymerase chain reaction (PCR).

**Results:**

a total of 1754 female An. gambiae s.l. were collected before and after deployment of ITNs. Fewer mosquitoes were collected after the distribution of ITNs. However, there was no significant difference in sporozoite rates or the overall entomological inoculation rate before and after the distribution of ITNs. Test-mosquitoes were resistant to deltamethrin, permethrin, and Dichlorodiphenyltrichloroethane but susceptible to bendiocarb. Pre-exposure of mosquitoes to Piperonyl butoxide increased their mortality after exposure to permethrin and deltamethrin. The frequency of the Kinase insert domain receptor (kdr)-West gene increased from 92 to 99% before and after the distribution of nets, respectively.

**Conclusion:**

seasonal impacts could be a limiting factor in the analysis of these data; however, the lack of decrease in transmission after the distribution of new nets could be explained by the high-level of resistance to pyrethroid.

## Introduction

Malaria remains one of the most important causes of mortality and morbidity in the Democratic Republic of Congo [1]. In 2015, 19,000,000 cases of malaria were recorded and 42,000 deaths [1]. In response to this important public health problem, the key strategy has been the control of the malaria vector using insecticide-treated bed nets (ITNs). They are effective in preventing vector bites because of the protective barrier provided by the net as well as the insecticide, which can kill or repel mosquitoes.

Currently, insecticide resistance of malaria vectors threatens to reduce the effectiveness of ITNs. For instance, resistance to pyrethroids has been reported in *Anopheles gambiae s.l*., a species complex that contains several major vectors of malaria in Democratic Republic of Congo (DRC) [2, 3]. Although there was no evidence of a loss of effectiveness of the insecticide, the prevalence of malaria in children using permethrin-treated nets was no different to those not using the ITNs (according to 2013-2014 survey by Demographic and Health Survey), which corresponds to the nation-wide resistance trends [4].

In addition to phenotypic resistance, the kdr-West mutation, which confers some resistance to pyrethroids and Dichlorodiphenyltrichloroethane (DDT), has been detected in Kingasani (Kinshasa), Bolenge (Equateur), Kimpese (Kongo Centrale) and Katana (Sud Kivu) [5]. The kdr-East mutation has also been detected in Kinshasa and Haut Uele provinces [6, 7]. Bandundu City (Kwilu) is located in an area of holo-endemic transmission, with members of the *Anopheles gambiae* complex as the primary vectors [8]. Two mass ITNs distribution campaigns were undertaken in 2007 and 2011, and the most recent campaign was conducted in December 2015. Studies carried out in many countries have reported an increase in insecticide resistance following such mass distribution campaigns, sometimes followed by a rebound in transmission [9-11]. The aim of this study was, therefore, to evaluate the susceptibility of malaria vectors to insecticides, identity the prevalence of insecticide resistance mechanisms, and record standard measures of parasite transmission before and after the mass distribution of ITNs in December 2015.

## Methods

**Figure 1:**
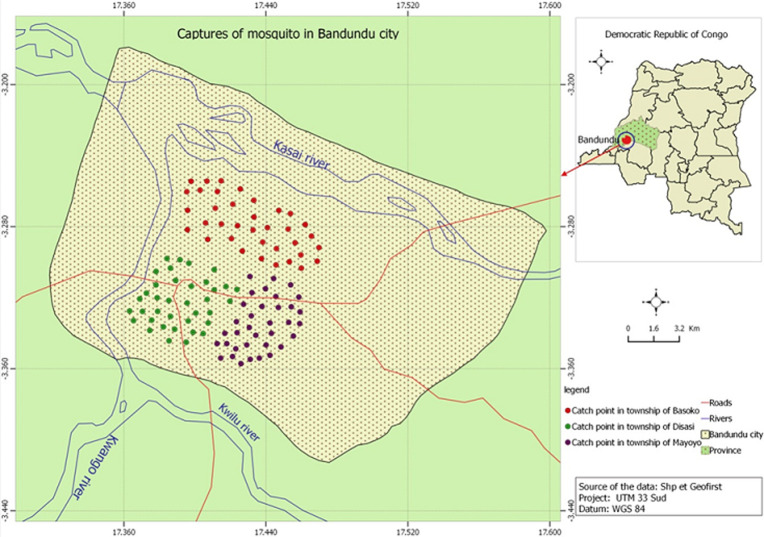
study site map of (circles indicate houses where mosquitoes where collected in three neighborhoods in Bandundu-City)

**Figure 2:**
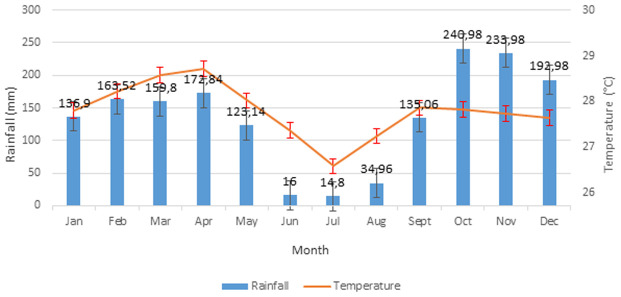
monthly temperature average and rainfall in Bandundu City, Kwilu Province, DRC, 2012-2016

**Study site:** Bandundu City (3.3168° S, 17.3790° E; 324m above the sea level) (-3.335123, 17.378747) is the capital of the province of Kwilu in the DRC. It is in a marshy area at the confluence of rivers Kwilu, Kwango and Kasai (Figure 1) and is approximately 400km away from the capital, Kinshasa. The population of the city, which includes three townships (Basoko, Disasi and Mayoyo), was estimated at 285,411 inhabitants in 2010 [12-14]. The average annual temperature is 26.9°C and is fairly constant throughout the year. The average annual rainfall is between 800 and 1500mm. Bandundu city experiences a humid tropical climate with two well-marked seasons: a long rainy season and a short (4-month) dry season, as shown in Figure 2 [13, 14]. A short dry period is often noted in January-February, followed by a short rainy period in March-April. Climatological data obtained from the meteorological department of Bandundu-City (METELSAT/BDD) is shown in Figure 2. The mass distribution campaign of ITNs (Dawa plus® 2.0, Tana Netting) was carried out between 17 and 29 December 2015.

**Mosquito collection:** a total of 108 houses were visited during the study from 15 July 2015 to 15 June 2016 (Figure 1). All sampled houses had mud walls and had tin or thatched roofs. Each month, nine houses were selected at random for mosquito sampling, with different houses selected for each monthly sampling event. Adult mosquitoes were collected between 06:00 and 10:00 am using pyrethrum spray catches [15]. All openings of the house were closed and white sheets were laid on the floor. A commercially available pyrethroid spray (Baygon, Bayer) was sprayed in the house and doors were closed for 15 minutes. The sheets were carefully removed from the house and inspected for mosquitoes, which were collected and placed individually into labelled tubes [15].

Human landing catches (HLCs) were done primarily to determine malaria vector species composition, the location of biting (indoors or outdoors) and times of biting. Mosquitoes were collected monthly from nine houses by two volunteers in six-hour shifts, from 18:00 h to 0:00 h and from 0:00 h to 6:00 h. Two collectors were posted inside the house in the living room and two outside the house, less than five meters from the front door. Different houses were used for each night. Each collector sat on a stool with his lower legs and feet exposed for mosquitoes to land on. The collector monitored mosquitoes as they landed and captured them with small glass tubes that were sealed with cotton wool. The latter were then placed in a sealed bag and labelled according to the hour of collection. These data were used to calculate the nightly human biting rate (HBR) based on eight person-nights of collection indoors and outdoors for each sampling period. The mosquito collectors for HLCs were recruited from the community and provided with requisite training. Collectors showing any signs of illness up to three weeks following collections were screened for malaria at a local health centre. There were no positive cases.

*Anopheles gambiae s.l*. mosquito larvae were collected from breeding sites and transferred to pans containing water from the site and were reared until adult stage in a field insectary. Larvae were not fed and survived from the nutrients in the site water. Adults were identified to species according to morphological identification keys [16, 17]. A subsample of mosquitoes identified as belonging to the *Anopheles gambiae* complex were identified to species as described below. The molecular identification and analysis of *An. gambiae s.l*. were performed at CDC (Atlanta, GA, USA).

**Laboratory analyses:** the species identification by PCR within the *An. Gambiae* complex was carried out according to the technique described by Wilkins *et al*.with a modified denaturation temperature of 94°C for 30 seconds and using 2X AccuStart II Gel Track PCR SuperMix according to manufacturer´s instructions (Quanta Biosciences). All mosquitoes were tested for the presence of the G119S mutation (ace-1), the presence of the L1014F mutation (kdr-West) and L1014S mutation (kdr-East) [18].

The PCR-based assays for all the three mutations were performed as described in the MR4 Methods in Anopheles Research Training Manual (section 5.3.5 and section 5.3.2) (MR4 2012). To confirm the results, both kdr-West and kdr-East-resistant individuals were sequenced using the following method: deoxyribonucleic acid (DNA) was amplified in a 50 µl reaction containing 2X AccuStart II GelTrack PCR SuperMix with 2 µM of primers AGD1 and AGD2 and 2 µl of DNA template [19]. Thermocycler conditions were an initial step of 94°C for 5 min followed by 35 cycles of 94°C for 30s, 48°C for 30s, 72°C for 30s, with a final elongation step of 72°C for 5 min. PCR products were observed on 1.5% agarose gel and purified using an Exo SAP mix to clean-up excess dNTPS and primers from the PCR reaction to prepare it for cycle sequencing for genetic analyses. The Exo SAP mix was prepared containing Exonuclease I (20 U/µl, New England BioLabs Cat. No. M0293S) with a final concentration of 0.050 U/µl and rAPid Alkaline Phosphatase (1 U/µl, Sigma-Aldrich Cat. No. 4898133001) with a final concentration of 0.025U/µl and 2.3µl of Exo SAP mix were added to 5µl of each PCR reaction.

Samples were incubated at 37°C for 30 min, followed by an incubation period of 5 min at 95°C in a Bio-Rad T100 Thermal Cycler and stored at -20°C until needed. The purified PCR products were diluted 1: 5 with water for cycle sequencing. One-quarter sequencing reactions were performed with Big Dye Terminator v1.1 and 1µl of the diluted purified PCR product for each reaction. Sequence reactions were purified with the Big Dye X Terminator Purification Kit (Life Technologies, Foster City, California, USA). The purified sequence reactions were run on a 3500xL Genetic Analyzer (Life Technologies, Foster City, California, USA) using the BDx Short module, according to the manufacturer´s instructions. The sequence data were analyzed using Seq Man Pro from the DNASTAR Laser gene 12 Core Suite (DNASTAR, Madison, Wisconsin, USA). To detect sporozoites in mosquitoes, heads and thoraxes were separated from abdomens according to the protocol described by Wirtz *et al*. [20].

**Entomological inoculation rates:** entomological inoculation rates (EIR) are used to estimate the risk of transmission by looking at the number of infectious bites people can be exposed to if prevention methods are not used. EIR pre- and post-ITN distribution were calculated by multiplying the proportion of mosquitoes found to be infective (sporozoite rate) by the average number of females collected by HLCs. The presence of nets prior to the distribution in December 2015 was not assessed. Similarly, net use after the distribution was not quantified.

**Insecticide susceptibility tests:** two to three-day-old female *An. gambiae s.l*., reared from immature stages collected in the field (see above), were tested for their susceptibility to different insecticides under ambient room temperature conditions (25-28°C and 70-80% relative humidity) using the standard WHO protocol [21]. All the tests for each insecticide were conducted the same day. Tests were conducted between 1-3 months before the ITNs distribution in December 2015 and 5-8 months after the ITN distribution).

The standard WHO insecticide susceptibility tests were conducted using test-kits and insecticide impregnated filter papers supplied by the WHO [20]. The insecticides used were pyrethroids (0.05% deltamethrin and 0.75% permethrin), an organochlorine (4% DDT) and a carbamate (0.1% bendiocarb). In addition to standard insecticide susceptibility tests, synergist bioassays were conducted for permethrin and deltamethrin by exposing mosquitoes to piperonyl butoxide (PBO) papers (5%) for 1 hour prior to conducting bioassays. These results were then compared with the insecticides without pre-exposure to PBO to evaluate the impact of oxidases on resistance.

Each test consisted of 25 mosquitoes per tube, with one control. Four replicates were used for each exposure set. For each bioassay, the knockdown effect of mosquitoes was recorded at regular intervals for 60 min and final mortality was recorded after a 24-h recovery period during which survivors were supplied with a 10% sugar solution. Insecticide susceptibility was classified according to WHO criteria, which consider mortality above 98% and below 90% to be representative of susceptible and resistant populations respectively, while populations showing intermediate mortality (between 90% and 97%) would require further investigation. Time to knockdown of 50% and 95% of mosquitoes (KDT50, KDT95) during the exposure period in each test was calculated. Dead and surviving mosquitoes were stored separately in labeled tubes containing silica gel for further analysis of the kdr mutation.

**Data analysis:** data were analysed using Epi Info 3.5.1 and then imported into Stata version 11.1 (Stata Corp., College Station, TX, USA). Normality of the data was assessed using the Kolmogorov-Smirnov test and visualized using a cumulative frequency curve of observed quantiles compared with theoretical quantiles [22]. The null hypothesis was in that which the normal distribution was rejected using p<0.05. The entomological parameters studied included the density of Anopheles, the biting rate, the sporozoite index, and the entomological inoculation rate (EIR).

The comparison and the analyses of entomological indices between the two periods (before and after the mass distribution of insecticide treated nets) were evaluated using the non-parametric Mann Whitney U test, due to the non-normality of the data. Chi-squared tests were used to assess proportional differences. A 0.05 cut-off was used in determining significance. The KDT_50_ and KDT_95_ were calculated using the probit analysis calculated with the Polo Plus program (LeOra Software, Petaluma, CA, USA) [23].

**Ethical approval:** this study was conducted with respect to the fundamental ethical principles as described in the Helsinki declaration and the protocol was approved by the political authorities of Bandundu City. All households provided informed consent prior to their inclusion in this study.

**Funding:** this work was supported by the University of Bandundu and INRB/Kinshasa. This study benefited from the technical support of the U.S. President´s Malaria Initiative. The team of researchers thanks VESTERGAARD for its financial support in carrying out this work.

## Results

**Entomological inoculation rates:** all the 930 specimens of *Anopheles* collected by PSC were *An. gambiae s.l*. All mosquitoes collected were tested for presence of circumsporozoite protein using ELISA. The results for each month of collection are shown in Table 1. Sporozoite rates ranged from 4.9 to 16.7%. Data from 824 specimens of *Anopheles* collected by HLCs were used to calculate the nightly human biting rate (HBR) based on nine person-nights of collection indoors and outdoors for each sampling period. Hourly data of Anopheles collected by HLCs were plotted on graphs to show biting time trends. The mean number of *An. gambiae s.l*. per house, the estimated number of bites per person per day and the entomological inoculation rates were highest in October during the long rainy season and lowest during the long dry season in the month of July (Table 1). However, the sporozoite index (SI) was not influenced by the rainy season (p=0.45). It was highest in February (short dry season) and lowest in May (beginning of the long dry season). It was observed also that the most of the *An. gambiae s.l*. were blood fed. The different entomological parameters of malaria transmission were evaluated before and after the distribution of ITN and are shown in Table 2.

**Table 1:** *Anopheles gambiae s.l*. mosquitoes collected in Bandundu between June 2015 and June 2016, including sporozoite rates

Year	Month	Houses sampled (PSC/HLCs)	Number of people in houses	Total number of *An. gambiae s.l*. collected HLCs PSC		Mean number of *An. gambiae s.l*./house	Total number of blood fed- *An. gambiae s.l*.	Estimated number of bites/night	*Anopheles gambiae s.l*. with sporozoites	Sporozoite rate	Number of infectious bites/person/day
2015	July	9	39	20	23	2.0	8	2.2	1	5.6	0.12
	August	9	65	68	73	7.3	51	7.5	5	7.6	0.52
	September	9	61	111	126	13.7	91	12.3	6	4.9	0.60
	October	9	44	220	206	26.1	145	24.4	37	15.7	3.8
	November	9	61	47	84	9.3	56	5.2	6	7.1	0.36
	December	9	53	39	48	6.3	40	4.2	7	12.3	0.51
2016	January	9	52	50	56	6.2	42	5.5	6	10.7	0.58
	February	9	46	42	54	5.3	26	4.6	8	16.7	0.76
	March	9	54	42	54	5.7	32	4.6	7	13.7	0.63
	April	9	47	20	21	2.0	11	2.2	2	11.2	0.24
	May	9	51	80	92	9.4	58	8.8	4	4.7	0.41
	June	9	52	85	93	9.9	60	9.4	8	9.0	0.8

**Table 2:** entomological parameters of malaria transmission before and after ITN distribution in December 2015

Parameters	Before distribution Jul – Dec 2015		After distribution Jan – Jun 2016		p-value
	entomological index	Median (IQR)	entomological index	IQR	
Relative density (mean numbers of *An. gambiae s.l*. collected)	10.8	5.00 (0-69)	6.4	4.00 (0-46)	0.365
Sporozoite Index (percentage of mosquitoes positive for CSP)	10.6	7.02 (0-100)	10.1	2.17 (0-50)	0.991
Biting Rate (*Anopheles gambiae s.l*. per person)	1.22	0.71 (0-8.33)	0.75	0.33 (0-4.5)	0.415
Entomological Inoculation Rate (EIR)(Number of infectious bites per person per day)	0.13	0.03 (0-1.71)	0.08	0.021 (0-0.33)	0.561

There was no significant difference (p = 0.365) between the numbers of *An. gambiae s.l*. per house before and after ITN distribution campaign: 10.8 An. gambiae s.l./house before the distribution vs. 6.4 after distribution. Furthermore, there were no significant differences between the numbers of *An. gambiae s.l*. per person, sporozoite indices, and entomological inoculation rates before and after ITN distribution campaigns (Table 2).

**Figure 3:**
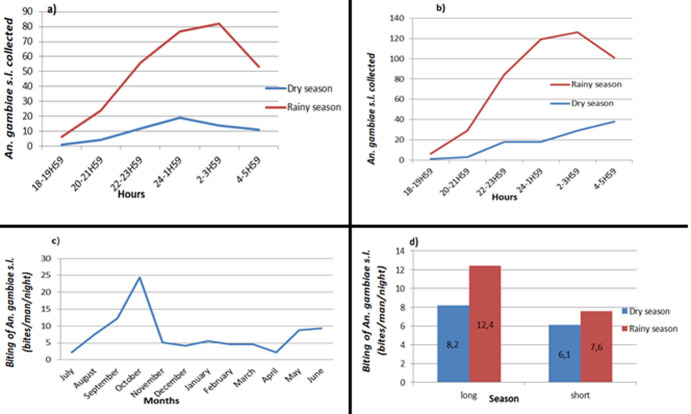
biting times of *An. gambiae s.l*. collected per HLC in July 2015-June 2016: (a) *An. gambiae s.l*. collected indoor per season; (b) *An. gambiae s.l*. collected outdoor per season; (c) year-round high *An. gambiae s.l*. biting rates; (d) *An. gambiae s.l*. biting rates per season

A total of 824 *An. gambiae s.l*. were collected using HLC, of which 465 were collected outdoor and 359 indoor. A change in behaviour of *Anopheles* mosquitoes was observed which became exophoric. The human biting rate (bites/man/night) varied over the seasons, with mosquito abundance notably higher during the main rainy season, peaking in October (Figure 3). The hourly activities of *Anopheles sp*. varied, with biting starting early at 18: 00-19: 00 hrs and gradually progressing to intensify around 24: 00-1: 00 hrs and reaching a second peak at 2: 00-3: 00 hrs (Figure 3).

For *An. gambiae s.l*. collected from July to December 2015, a significant difference (p <0.01) was observed between all the entomological indices (HBR, density, EIR and SI) during the dry season and the rainy season (Table 3). Entomological indices of transmission were significantly influenced by seasons, except Anopheles density during the second phase of January-June 2016 where no significant difference was observed (p = 0.24). By comparing these two capture phases, no significant difference was observed between aggressiveness, sporozoite index and entomological inoculation rate, with p=0.713, p=0.098 and p=0.896, respectively. The seasonal variations between these two collection phases were not significant.

**Table 3:** distribution of entomological indices of malaria transmission by season

Parameters	Dry season		Rainy season		p-value
	entomological index	Median (IQR)	entomological index	Median IQR	
Relative density (mean numbers of *An. gambiae s.l*. collected)	7.67	3.00 (1.3-6)	15,8	13.00 (10-16.7)	**0.01**
Sporozoite index (percentage of mosquitoes positive for CSP)	6.03	0.00 (0-0.076)	11,7	0.029 (0-0.069)	**0.01**
Biting Rate (*Anopheles gambiae s.l*. per person)	2.8	0.46 (0.3-1)	9,1	2.4 (1.3-4)	**<0.001**
Entomological Inoculation Rate (EIR) (Number of infectious bites per person per day)	0.16	0.00 (0-0.041)	1,22	0.082 (0-0.148)	**<0.001**

**Insecticide susceptibility:** all *An. gambiae s.l*. tested were resistant to DDT, permethrin, and deltamethrin before and after the ITN distribution (Table 4). Resistance to DDT was particularly strong as mortality was never greater than 16%. Similarly, permethrin mortality ranged from 31 to 36%. Mortality following exposure to deltamethrin was 52% in the first bioassay and 34% in the second bioassay.

**Table 4:** insecticide susceptibility, expressed as KDT_50_, KDT_95_, and 24 hour mortality, of *Anopheles gambiae s.l*. collected from Bandundu City before and after ITN distribution in December 2015

Period	Insecticide	N	KDT_50_ (minutes)	KDT_95_ (minutes)	Mortality % (24h)	Conclusion
Before ITN distribution (September-November 2015)	Deltamethrin	100	42.6 (40.7-44.8)	n/a	52	Resistant
	Deltamethrin+PBO	100	22.7 (21.5-23.7)	39.7 (37.0-43.3)	98	Susceptible (Full involvement of P450 oxidase mechanisms)
	Permethrin	100	n/a	n/a	31	Resistant
	Permethrin+PBO	100	66.6 (61.7-74.2)	n/a	84	Resistant (Partial involvement of P450 oxidase mechanisms)
	Bendiocarb	100	27.7 (25.5-29.8)	43.2 (39.1-50.3)	100	Susceptible
	DDT	100	n/a	n/a	4	Resistant
After ITN distribution (May-August 2016)	Deltamethrin	100	31.5 (30.4-32.5)	51.7 (49.2-54.9)	34	Resistant
	Deltamethrin+PBO	100	30.9 (29.5-32.3)	52.1 (48.6-56.8)	99	Susceptible (Full involvement of P450 oxidase mechanisms)
	Permethrin	100	n/a	n/a	36	Resistant
	Permethrin+PBO	100	67.9 (61.9-77.2)	n/a	97	Probably resistant (Partial involvement of P450 oxidase mechanisms)
	Bendiocarb	100	18.6 (17.3-19.8)	31.5 (28.9-35.4)	100	Susceptible
	DDT	100	n/a	n/a	16	Resistant

The addition of PBO resulted in the restoration of susceptibility to deltamethrin both before and after the ITN distribution but at a lesser extent with permethrin. For instance, mortality after exposure to permethrin was 31% without PBO but 84% with PBO prior to the distribution of ITNs. On the other hand, mortality increased from 36% without PBO to 97% with PBO following the distribution of ITNs. The susceptibility of Anopheles to bendiocarb was 100% before and after distribution of ITNs (Table 4).

**Figure 4:**
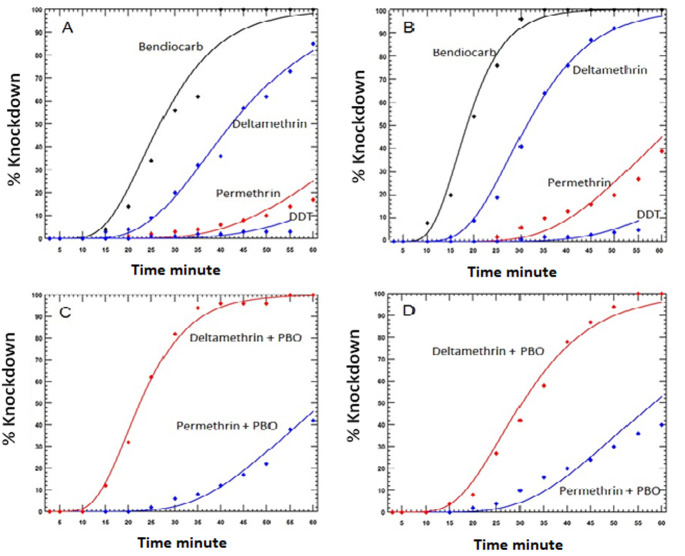
mortality of *Anopheles gambiae s.l*. in WHO susceptibility tests conducted: (A) before ITN distribution, without synergists; (B) after ITN distribution, without synergists; (C) before ITN distribution with synergists; (D) after ITN distribution, with synergists

When the speed of knockdown of the different insecticides was evaluated, bendiocarb had the fastest knockdown effect, followed by deltamethrin, permethrin, and finally DDT (Figure 4). There was a slight increase in the speed of knockdown for deltamethrin and permethrin when PBO was added prior to the ITN distribution. The knockdown times for both insecticides were similar with and without PBO the after the ITN distribution, although the 24-hour mortalities were different.

**Resistance mechanisms:** there was a high frequency of the kdr-West mutation (L1014F) before and after the distribution of ITNs (Table 5). The frequency of kdr-West in *An. gambiae s.s*. was 92% and 99%, before and after the distribution of ITNs, respectively. *Anopheles coluzzii* was found in small numbers prior to the distribution of ITNs (n=5) and had a high frequency of kdr-West. In addition to kdr-West, small numbers of kdr-East were detected in *An. gambiae s.s*. (4% at both time points). Of interest were 257 specimens, which had both the kdr-West and kdr-East mutations. No acetyl cholinesterase resistance mechanism gene was identified in *An. gambiae s.s*. in the study site. Mass distribution of ITNs significantly increased the risk of resistance emergence of kdr-West genes with OR 8.6 (1.1-70.2) (p = 0.02).

**Table 5:** presence of kdr-West and kdr-East mutations in identified subsample of *Anopheles gambiae* complex mosquitoes before and after an ITN distribution

Period	Species	n	L1014F (kdr-West)				L1014S (kdr-East)			
			RR	RS	SS	Frequency (%)	RR	RS	SS	Frequency (%)
October 2015	*An. gambiae s.s*.	151	130	18	3	92	3	0	148	4
	*An. coluzzii*	5	4	1	0	90	n/a	n/a	n/a	n/a
June 2016	*An. gambiae s.s*.	101	99	2	0	99	2	0	99	4

## Discussion

The transmission of malaria is prevalent and year-round in Bandundu City and therefore requires an effective vector control [1, 8]. Vector control is the pillar of malaria control and relies primarily on the distribution of ITNs and the implementation of indoor residual spraying (IRS). Both interventions are being used in the DRC, but IRS is done on a limited scale by private companies. The National Malaria Control Program (NMCP) adopted mass distribution of ITNs as a malaria control strategy in 2004, as a tool in interrupting malaria transmission [1, 2]. Since then, the NMCP has distributed millions of ITNs treated with pyrethroids. However, there are few data available on the insecticide resistance of Anopheles in DRC, though the emergence of insecticide resistance has the potential to impact the efficacy of vector control interventions [24-27]. This study, therefore, aimed at evaluating mosquito resistance to insecticides and the main entomological indicators associated with malaria transmission before and after ITN distribution in Bandundu City.

**Entomological inoculation rates:** the distribution of ITNs in Bandundu City in December 2015 resulted in a decrease in the numbers of *Anopheles* mosquitoes collected in households by pyrethroid spray catch and Human landing catch techniques, although the difference was not significant. Similarly, no significant differences were found between sporozoite indices, biting rates, or entomological inoculation rates (EIR) between the two periods. The risk of infective bites prior to the bed net distribution was approximately 0.13 infective bites per person per night, equivalent to 47.2 infective bites per person per year while it was 0.08 infective bites per person per night or 27.6 infective bites per person per year after the distribution of ITNs. However, this decrease was not significant.

The distribution of ITNs resulted in the decrease in the number of Anopheles mosquitoes. However, it is difficult to attribute this decrease to LLINs. There was an obvious influence of the season on entomological indices of transmission which were high during the rainy season and low in the dry season. In addition, there is the distribution of two seasons, the rainy season (September-December and April-May) and the dry season (June-August and January-March) which makes it still difficult to assess the decrease in indices entomological. A high rate of mosquito bites was recorded outdoors in Bandundu-city during the collection campaign, suggesting behaviour changes of *An. gambiae s.l*. as reported elsewhere [28, 29]. However, it is difficult to attribute the change of the behaviour to the only repellent effect of deltamethrin-treated LLINs [28, 29]. A habit was observed among the population who stayed outside late at night to watch television soaps. This may explain why the peak of anopheline activity was reached between 2-3 am.

The detection of five specimens carrying both the kdr-East and kdr-West confirms the combination of kdr mutations as reported elsewhere in DRC [6, 7, 30]. These results are different from the ones reported in South Africa, where control using IRSfailed [31]. In Dielmo, an area where pyrethroids were the primary insecticides used for the control of mosquito malaria, a rebound in the number of malaria cases was recorded, followed by the development of resistance after multiple ITNs distributions. [32]. In another study in Benin, a reduction in the efficacy of ITNs was observed, resulting in increase of malaria cases in an area with pyrethroid-resistant *Anopheles gambiae* and deltamethrin treated nets [33].

There were a number of limitations in this study which should be taken into account while interpreting the results. First, ELISA was the method used to determine sporozoite positive mosquitoes, which sometimes may result in false positives [34]. This shortcoming could be addressed by heating the ELISA lysate to 100°C for 10 minutes, but this was not done. Secondly, the collection of mosquitoes was made using pyrethrum spray catches, so determining sporozoite. As shown in Table 1, considerable number of non-fed mosquitoes was collected from the houses, which might have played a role in malaria transmission by feeding later. These results therefore possibly underestimate the number of bites per person and might have resulted in bias when new ITNs were deployed, resulting in more mosquitoes exiting before they could be collected. Moreover, blood-fed mosquitoes were not tested for human blood, although it might have been assumed that most blood-fed *An. gambiae s.l*. resting indoors would have fed on humans. Thirdly, since mosquito collections for pre-net distribution and post-net distribution were carried out during different months of dry and rainy seasons, seasonal variations cannot be ruled out in interpreting collection and bioassay data.

Metelo *et al*. reported seasonal differences in *An. gambiae s.l*. populations in Bandundu between dry and rainy seasons [35]. It should be noted that in many settings, nets are not used immediately upon their receipt, but rather after the old nets are torn and no longer usable. The use of the newly distributed nets in the houses was not quantified, although the presence of Dawa Plus nets was observed in most of the households during the execution of the PSCs and HLCs. Large-scale use of DHS data have shown that ITN use is still associated with reduced malaria transmission, especially when community use is high, however, insecticide resistance may be reducing this effect, Ferrari *et al*. found that sleeping under an ITN the previous night was associated with reduced risk of *Plasmodium* infection [36, 37]. In the present study, however, there were no high levels of impact on entomological measures of transmission immediately following the distribution of ITNs. In an area with already high levels of insecticide resistance, the distribution of new ITNs does no longer have an immediate or strong effect on the main entomological measures of malaria transmission. This may be due to an increased resistance in the study area, compromising both new nets with a full dose of insecticide, and the old nets, which will have lost some of their insecticide. It may also mean that the old nets remained effective through the three years expectance of the net, and so the distribution of new nets did not result in improved control. However, the presence of sporozoite-positive mosquitoes in both periods indicates that improved control measure is needed to reduce transmission in this area.

**Resistance to pyrethroids and DDT:** except bendiocarb which caused 100% mortality of Anopheles mosquitoes, the other tested insecticides were ineffective for *An. gambiae s.l*. collected before and after the distribution of ITNs. *An. gambiae s.l*. was resistant to pyrethroids (deltamethrin and permethrin) and DDT during both periods. The mortality of Anopheles to insecticides varied according to the period (before and after mass distribution of ITNs). Mortality was limited to deltamethrin, 52% before mass distribution and was reduced to 34% after, which reduced the efficacy of this product. After pre-exposure to PBO, the efficacy of deltamethrin was fully restored during the 2 study periods. For permethrin (31-36%) and DDT (4-16%) Anopheles mosquitoes were also resistant to varying degrees depending on the period (before-after). We observe that after the mass distribution ITNs, permethrin and DDT have increased their efficacy a little. This could be explained by the fact that distributed LLINs were impregnated with deltamethrin which increased the selective pressure and served as the basis for the emergence of resistance of *An. gambiae s.l*. This resistance poses fundamental and operational problems to the extent the behaviour of Anopheles is modified, resulting in significant decrease in the effectiveness of these products. The endemicity of malaria and the high number of infected *An. Gambiae* in the city of Bandundu raise concern and should be addressed.

## Conclusion

In an area with high insecticide resistance, no significant decreases in entomological parameters (numbers of mosquitoes collected per night, sporozoite rates) were observed following mass distribution of ITNs. Factors such as seasonal mosquito population dynamic or resistance status, bed net usage, and other factors affecting malaria transmission should be further evaluated. The level of insecticide resistance observed in Bandundu City is of concern for malaria control in the DRC. Since the emergence of resistance is related to one class of insecticide (pyrethroids) only (recommended for the impregnation of mosquito nets in mosquito vectors), the issue of their repellence and lethality is of concern. Hence, there is the need to consider new alternatives to curb the emergence of this resistance and maintain the gains achieved so far achieved.

### What is known about this topic

- The resistance of the An.gambiae s.l.population in Bandundu City to pyrethroids (permethrin and deltamethrin) is known and this resistance is significantly improved by the use of PBO;
- The efficacy of mass use of ITNs has been proven in malaria endemic areas;
- Malaria transmission is highly influenced by seasonality.

### What this study adds

- The resistance of the An. gambiae s.l.population in Bandundu City to pyrethroids (permethrin and deltamethrin) and DDT is very high;
- The mass distribution of ITNs in Bandundu City did not lower the entomological indices of malaria transmission;
- The rainy season has significantly influenced the entomological indices of malaria transmission.
